# 1174. Impact of an Electronic Antibiotic Time-out Best Practice Alert on Antibiotic Use and Prescribing Behavior in Hospitalized Patients

**DOI:** 10.1093/ofid/ofad500.1014

**Published:** 2023-11-27

**Authors:** Tyler Ackley, Joseph L Kuti, Anastasia Bilinskaya, Kristin E Linder, Casey J Dempsey

**Affiliations:** Hartford Hospital, Hartford, Connecticut; Hartford Hospital, Hartford, Connecticut; Hartford Healthcare, Hartford, Connecticut; Hartford HealthCare, Hartford, Connecticut; Hartford Healthcare, Hartford, Connecticut

## Abstract

**Background:**

Antibiotic time-outs (ATOs) are an evolving stewardship strategy capable of widespread impact with relatively low perceived personnel effort. ATO-based interventions have been shown to improve the rates of antibiotic de-escalation and increase the likelihood of antibiotic optimization. In August 2022, Hartford HealthCare implemented a 72-hour ATO best practice alert (BPA) within the electronic medical record (EMR). Herein, we evaluated the impact of this intervention on antibiotic use and describe behaviors associated with alert prompting.

**Methods:**

This is a multi-center, quasi-experimental, retrospective chart review of admitted patients receiving empiric antibiotics for > 72 hours. An ATO alert was designed and embedded within the EMR and set to fire between the hours of 0700 and 1630. On BPA firing, prescribers were prompted to assess for antibiotic modification – a composite including discontinuation, de-escalation, inclusion of stop-date, and transition to oral therapies. Differences in the rate of antibiotic modification and overall antibiotic prescribing patterns for the 10 most frequently utilized antibiotics were compared between a pre-implementation control period (10/1/21 – 10/31/21) and a post-implementation period (10/1/22 – 10/31/22).

**Results:**

A total of 800 patients were included for analysis. There was no significant difference in the rate of antibiotic modification when comparing the pre- and post-implementation cohort (54.5% vs 57.5%, p = 0.433); however, there was a numerically lower rate of antibiotic escalation in the post-cohort (9.5% vs 5.8%, p = 0.062). The duration of antibiotic therapy was longer in the post-implementation cohort (4.7 vs 5.0 days, p < 0.001).

**Conclusion:**

Despite ATO implementation, rates of antibiotic modification were similar between the pre- and post-cohort. Further work to understand the optimal approach to implementing an ATO will be required to improve the impact on antimicrobial therapy.

**Disclosures:**

**Joseph L. Kuti, PharmD**, bioMeriuex Inc.: Grant/Research Support|Entasis Therapeutics: Grant/Research Support|Merck & Co, Inc: Grant/Research Support|Shionogi Inc: Advisor/Consultant|Shionogi Inc: Grant/Research Support|Shionogi Inc: Honoraria

